# The Distribution of Skull Score and Skull Density Ratio in Tremor Patients for MR-Guided Focused Ultrasound Thalamotomy

**DOI:** 10.3389/fnins.2021.612940

**Published:** 2021-05-17

**Authors:** Kevin Wen-Kai Tsai, Jui-Cheng Chen, Hui-Chin Lai, Wei-Chieh Chang, Takaomi Taira, Jin Woo Chang, Cheng-Yu Wei

**Affiliations:** ^1^MR-guided Focused Ultrasound Center, Chang Bing Show Chwan Memorial Hospital, Changhua City, Taiwan; ^2^Neuroscience Laboratory, Department of Neurology, China Medical University Hospital, Taichung City, Taiwan; ^3^School of Medicine, College of Medicine, China Medical University, Taichung City, Taiwan; ^4^Department of Neurology, China Medical University Hsinchu Hospital, Hsinchu, Taiwan; ^5^Department of Neurosurgery, Chang Bing Show Chwan Memorial Hospital, Changhua City, Taiwan; ^6^Department of Neurosurgery, Tokyo Women’s Medical University, Tokyo, Japan; ^7^Department of Neurosurgery, Yonsei University College of Medicine, Seoul, South Korea; ^8^Department of Exercise and Health Promotion, College of Kinesiology and Health, Chinese Culture University, Taipei, Taiwan; ^9^Department of Neurology, Chang Bing Show Chwan Memorial Hospital, Changhua City, Taiwan

**Keywords:** tremor, skull density ratio, skull score, MR-guided focused ultrasound thalamotomy, essential tremor

## Abstract

**Objective:**

Magnetic resonance-guided focused ultrasound (MRgFUS) is a minimum-invasive surgical approach to non-incisionally cause the thermos-coagulation inside the human brain. The skull score (SS) has already been approved as one of the most dominant factors related to a successful MRgFUS treatment. In this study, we first reveal the SS distribution of the tremor patients, and correlate the SS with the image feature from customized skull density ratio (cSDR). This correlation might give a direction to future clinical studies for improving the SS.

**Methods:**

Two hundred and forty-six patients received a computed tomography (CT) scan of the brain, and a bone-enhanced filter was applied and reconstructed to a high spatial resolution CT images. The SS of all patients would be estimated by the MRgFUS system after importing the reconstructed CT images into the MRgFUS system. The histogram and the cumulative distribution of the SS from all the patients were calculated to show the percentage of the patients whose SS lower than 0.3 and 0.4. The same CT images of all patients were utilized to calculated the cSDR by first segmented the trabecular bone and the cortical bone from the CT images and divided the average trabecular bone intensity (aTBI) by the average cortical bone intensity (aCBI). The Pearson’s correlations between the SS and the cSDR, aTBI, and the aCBI were calculated, respectively.

**Results:**

There were 19.19 and 50% of the patient who had the *SS* lower than the empirical threshold 0.3 and 0.4, respectively. The Pearson’s correlation between the SS and the cSDR, aCBI, and the aTBI were *R* = 0.8145, 0.5723, and 0.8842.

**Conclusion:**

Half of the patients were eligible for the MRgFUS thalamotomy based on the SS, and nearly 20% of patients were empirically difficult to achieve a therapeutic temperature during MRgFUS. The SS and our cSDR are highly correlated, and the SS had a higher correlation with aTBI than with aCBI. This is the first report to explicitly reveal the SS population and indicate a potential way to increase the chance to achieve a therapeutic temperature for those who originally have low SS.

## Introduction

High intensity focused ultrasound (HIFU) is an incisionless surgical device that has been widely used in medical research and clinical trials including the treatment of tumors (Kennedy, 2005) such as the liver (Kennedy et al., 2004; Illing et al., 2005), and kidney for over 50 years (Kennedy et al., 2003). By using the heating or cavitation at a variable distance from the transducer, HIFU can cause selectively thermal coagulation in a well-defined volume.

Sharing similar principles of the HIFU, recently the trans-cranial magnetic resonance-guided focused ultrasound (MRgFUS) has been widely used in different clinical trials to treat various neurological disorders in the human brain (Elias et al., 2016; Leinenga et al., 2016). MRgFUS can perform a thermal ablation around the subcortical area or opening the blood-brain barrier (Abbott et al., 2006) for drug delivery with different ultrasound frequency. One of the important keys to these successes was the phase aberration correction (Fry and Barger, 1978; Sun and Hynynen, 1998; Hynynen et al., 2004) across thousands of the ultrasound beams when passing through the skull (Chang et al., 2016; Jung et al., 2019) that produce a constructive focusing at the target area which maximize the energy-heat efficiency without causing any thermal coagulation outside the target. A high spatial resolution (<2 mm slice thickness) of a volumetric computed tomography (CT) scan with bone-enhanced filtering was used not only to delineate the entire skull for the phase aberration correction during the treatment but also to estimate a general skull score (SS) to screen patients before the treatment. The SS, which is offered by the InSightec, directly correlated with the temperature efficiency in the MRgFUS thalamotomy (Chang et al., 2016), and the empirical criterion of the SS for the MRgFUS thalamotomy is 0.40 (Wang et al., 2018). However, no literature was reported regarding the percentage of patients who can feasibly receive MRgFUS thalamotomy under this empirical criterion.

In this study, we tried to reveal the population distribution of the SS in tremor patients of Taiwanese people to address the aforementioned question, and report on the relationship between the SS and a customized skull density ratio (cSDR) by our algorithm, to determine the reliability of cSDR and the importance of the trabecular bone or the cortical bone separately in bone density calculation to provide fundamental information before designing a solution for low SS in MRgFUS treatment.

## Materials and Methods

### Patient Selection

Two hundred and forty-six patients (62.4 ± 12.0 years old, ranging from 23 to 89 years old, 162 male and 84 female) with tremor dominant symptoms visited our site for the MRgFUS thalamotomy screening and were recruited in this study. Enrolled patients gave informed consent and were diagnosed by neurologists specializing in movement disorders based on tremor criteria (Wang et al., 2018). The experiments conformed to the standards set by the Declaration of Helsinki and were approved by the Chang Bing Show Chwan Memorial Hospital, Taiwan. Conventional CT scans were arranged for those who were willing to join the MRgFUS thalamotomy screening with no unnecessary radiation exposure.

### Conventional CT Scan

A sixteen-slice CT scanner (LightSpeed, GE, United States) was used to acquire the brain CT image. The scanning parameters followed the head routine scan protocol in our hospital and they were: peak X-ray tube voltage = 120 kVp, tube current = 250 mA, and scan time = 1.0 s. The scanning slices were parallelly aligned with the orbital-meatus (OM) line by the visual check of a radiographer. All the projection images of respective patients were used to reconstruct a volumetric image with a slice thickness of 0.625 mm. A high-pass filter (Bone + filter) was applied to the reconstructed CT images to enhance the dynamic range of the skull while suppressing that of the other parts in the images.

### SS Calculation

**FIGURE 1:**
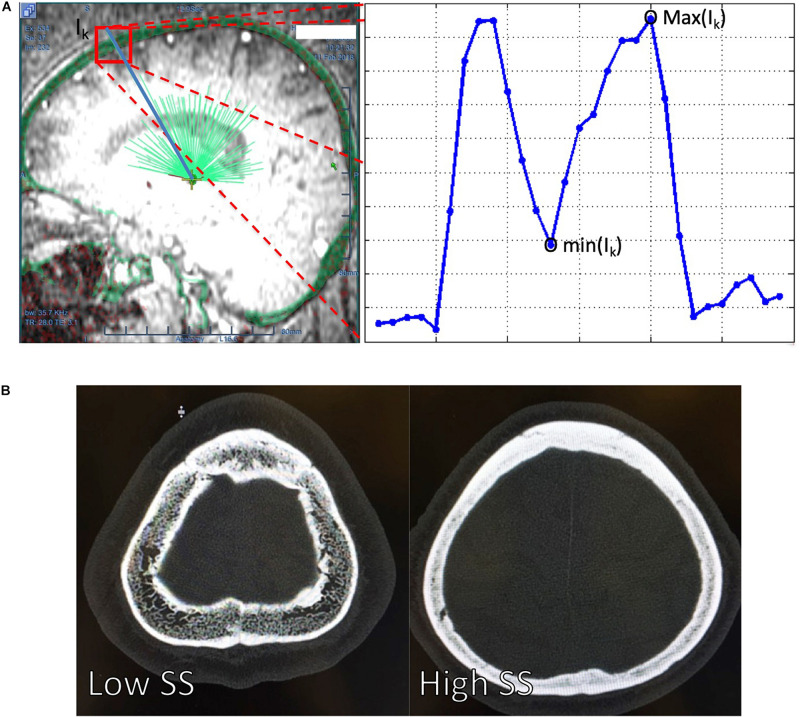
**(A)** The ExAblate Neuro platform simulated the ultrasound beams (light green lines) penetrating through a skull (transparent green around the head) and extracted the maximum [max (*I*_*k*_)] and minimum [min(*I*_*k*_)] of the skull intensities along each of the ultrasound. The blue line at the right panel of the **(A)** delineated the CT image intensity changes along one of the ultrasound beam *I*_*k*_. A high intensity difference between and cortical and trabecular bone might leaded to a low SS = 0.25 [**(B)**, left] compared to high SS = 0.70, which had a relatively similar intensity between the cortical and trabecular bone [**(B)**, right]. SS, skull score.

The CT images of each patient were uploaded into the ExAblate Neuro platform (InSightec Inc., Israel) and followed the procedures suggested by InSightec to calculate the SS. Specifically, the volumetric CT images with high-pass filtering of each patient were first uploaded into the ExAblate Neuro platform, and the anterior commissure, posterior commissure, and mid-sagittal point were defined on the platform. We used the built-in tool named target defined by AC-PC in the ExAblate Neuro platform to simulate a target for the MRgFUS thalamotomy. The spatial position of a simulated MRgFUS transducer helmet was then adjusted to align the ultrasound transducer focus and the simulated target of each patient. After the target selection, the calcification of each patient on the CT images was manually marked to turn off the ultrasound transducers which routes to the target pass through the marked calcification. After the brain calcification selection, the treatment protocol will be selected and the fiducial will be manually placed. In the treatment tag of the ExAblate Neuro platform, the SS from respective patients were reported from the ExAblate Neuro platform. Figure 1A showed how the ExAblate Neuro platform simulated the ultrasound beams penetrated through the skull, and the definition of the maximum and minimum values of the skull along the *I*_*k*_-th ultrasound beam. The SS for each patient was calculated by Eq. (1),

(1) $$\text{SS}=\frac{\sum_{k=1}^{p}\text{min}\,\left(I_{k}\right)/M\,a\,x\,\left(I_{k}\right)}{P}$$

where *P* indicated the number of ultrasound beams from the transducer.

### cSDR Calculation

The same CT images of each patient were used to calculate the SS ratio by our method. Specifically, we first used the image contrast window and level provided by a high-pass filter to enhance the cortical and trabecular bone and used Otsu’s method (Otsu, 1979) to separate the bone from the non-bone objects in the volumetric CT image. A mask volume was created by setting the intensity of bone to unity and that of non-bone to zero. The middle slice of the mask volume was used as the initial slice for the following analysis. Repeated image erosion was performed on the initial slice, and stopped before all the binary voxels were eroded. One of the remaining voxels was randomly selected and used as the seed point for the region growing on the initial slice. The candidates of the seed points for region growing of the adjacent slices to the previous slice were selected from the overlap between two adjacent slices to ensure spatial continuity. The region growing procedure was stopped until all the slices of the CT mask image were performed. The mask image from the results after region growing was used to define the entire skull. The aforementioned procedure to the volumetric CT image was repeatedly performed for 30 times by considering the balance between the computation time and the accuracy of the skull definition. All the 30 masked images were added together into one masked image, with the voxel values ranging from 0 to 30. We used the part of the skull where the masked image value equals 30, indicating the part most spatially continuous from the initial slice, to exclude the part of the bone that might be less related to the regions of the MRgFUS thalamotomy, such as maxillary bone. The Outs’ method was again used to obtain the image intensity threshold of the cortical bone and trabecular bone of the skull. The SDR (Eq. 2) was calculated by dividing the average trabecular bone intensity (aTBI) across all N voxels by the average cortical bone intensity (aCBI) across all *M* voxels.

(2) $$a\,T\,B\,I=\frac{\sum_{i=1}^{N}T\,B\,I_{i}}{N},a\,C\,B\,I=\frac{\sum_{j=1}^{M}C\,B\,I_{j}}{M},\text{c}\,S\,D\,R=\frac{a\,T\,B\,I}{a\,C\,B\,I}$$

All the aforementioned calculations were performed on the MATLAB (MathWorks, United States) platform.

### SDR Calculation

The histogram of all SSs from 246 patients was computed with the bin range from 0.1 to 0.8 and a step size of 0.01. The cumulative distribution function (Park, 2018) of the SS was computed from each bin of the histogram, leading a range from 0.1 to 0.8 and step size of 0.01.

### Correlation Between SS and cSDR

The linear correlations between the SS and the cSDR across all the patients were estimated by calculating the Pearson’s correlation coefficient between the SS and the cSDR from each of the patients. Specifically, the bootstrap sampling (Efron, 1979) was applied to produce 24,600 pairs of SS-cSDR pairs by repeatedly selecting 246 samples from our 246 data. For example, one can select [1,1,1,2,2,3,3,3,…, 122] or [1,2,3,4,5,6,…,246] from our 246 data while the number here in the bracket means the data index. A new SS-cSDR pair was produced by averaging across the SS and cSDR of each of the selections. The data selection and averaging were repeated 24,600 times, then we had 24,600 SS-cSDR pairs. The linear correlation between the SS and the cSDR after bootstrap sampling was estimated by calculating the Pearson’s correlation coefficient across all the bootstrapped pairs. Similarly, the linear correction of the SS-aCBI and SS-aTBI pairs across all the patients was respectively estimated to evaluate the influence of the aCBI and the aTBI on the SS. The correlation coefficient of the aforementioned calculation was done on MATLAB (MATLAB and Statistics Toolbox Release 2018a, The Math Works, Inc., Natick, MA, United States) by using the *corrcoef* function. The Pearson’s correlation coefficient and the *p*-value of this calculation were reported accordingly.

## Results

The 246 patients’ demographic characteristics are summarized in Table 1. There were 114 clinically diagnosed essential tremor (ET) patients, and the rest of them experiencing other types of tremors, including Parkinson’s disease and psychogenic tremor, etc. Figure 1B shows an exemplar of the trans-axial CT images with low (SS = 0.25) and high (SS = 0.70). The main difference between the CT images with the low (Figure 1B, left) and the high SS (Figure 1B, right) was that the contrast between the cortical and trabecular bone was higher in the low SS CT image than that in the high SS CT image. According to Eq. (1), a high intensity difference between the cortical and trabecular bone would lead to a high SS.

**TABLE 1:** Demographic characteristics in subjects with tremor.

Variables	Number (%)	Mean ± SD	*p*-value
Female/male	83/163 (33.7/66.3)		
Age (years)		62.41 ± 12.04	
ET/non-ET	192/54 (78/22)		
SS (total)		0.41 ± 0.12	
SS (female)		0.40 ± 0.12	
SS (male)		0.41 ± 0.12	0.88^1^
SS (ET)		0.41 ± 0.12	
SS (non-ET)		0.41 ± 0.13	0.66^2^

*ET, essential tremor; non-ET, non-essential tremor; SS, skull score. ^1^Comparision of SS between female and male. ^2^Comparision of SS between ET and non-ET.*

### SS Distribution Calculation

**FIGURE 2:**
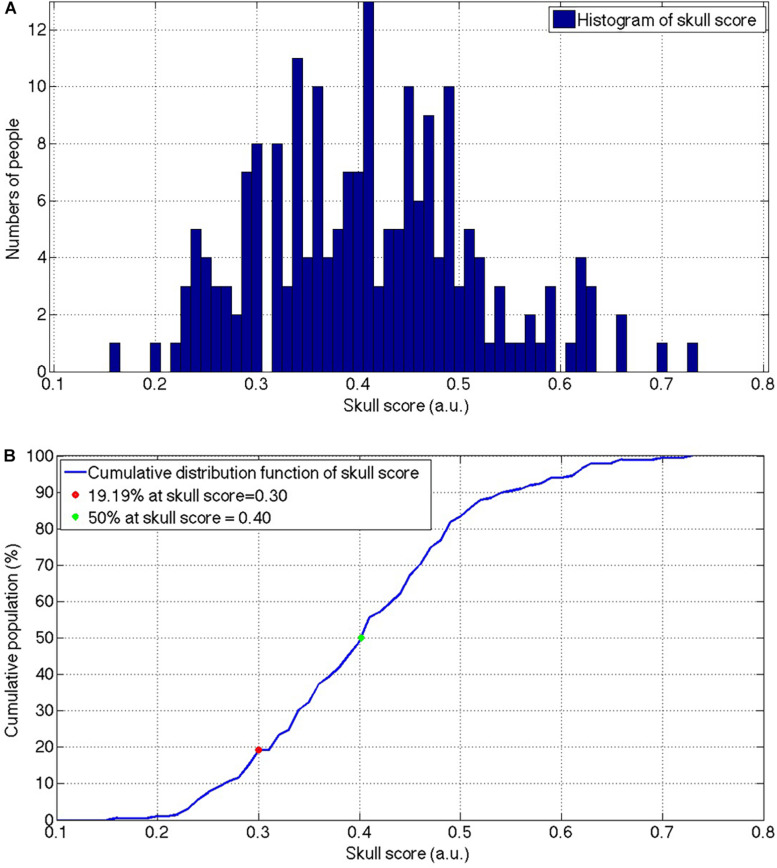
The histogram **(A)** and the cumulative distribution function **(B)** of the SS for the patients who received the MRgFUS thalamotomy screening. The envelope of the histogram showed a slightly positive skewed distribution and the peak was located at 0.41. The slope of the cumulative population was visually consistent from the beginning and start to decrease after the SS = 0.50. SS, skull score; MRgFUS, Magnetic resonance-guided focused ultrasound.

The mean and the standard deviation of the SS from the total 246 patients were 0.419 ± 105 and ranged from 0.16 to 0.73. Figure 2A shows the histogram of the SS of the included patients and there was a population peak at the SS = 0.41. Figure 2B shows the cumulative distribution function of the SS.

### Correlation Between SS and cSDR

**FIGURE 3:**
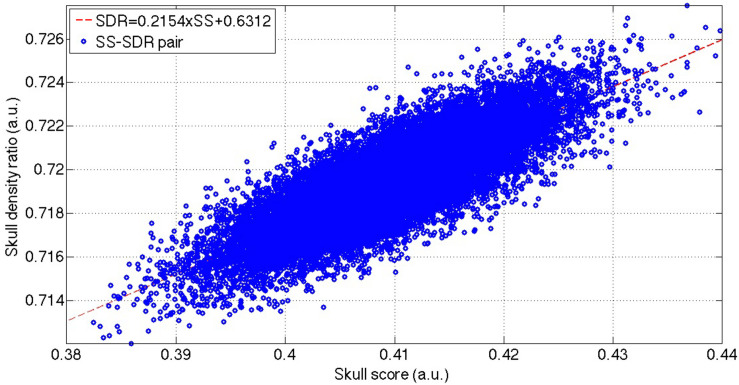
The distribution of the bootstrapped SS-cSDR pairs and the linear correction between these SS-SDR pairs. The blue dots show the total 2,46,000 bootstrapped samples, and the red line indicates the linear fitting of these bootstrapped samples. SS, skull score; cSDR, customized skull density ratio.

The total 2,46,000 bootstrapped samples (Figure 3) resulted in the SS ranging from 0.38 to 0.44 and cSDR ranging from 0.712 to 0.728. The Pearson’s correlation coefficient between the SS and cSDR was 0.8145 with the statistical significance (*p* < 10^–5^), and linear relation between the SS and cSDR:

(3) S⁢D⁢R=0.2154×S⁢S+0.6312

**FIGURE 4:**
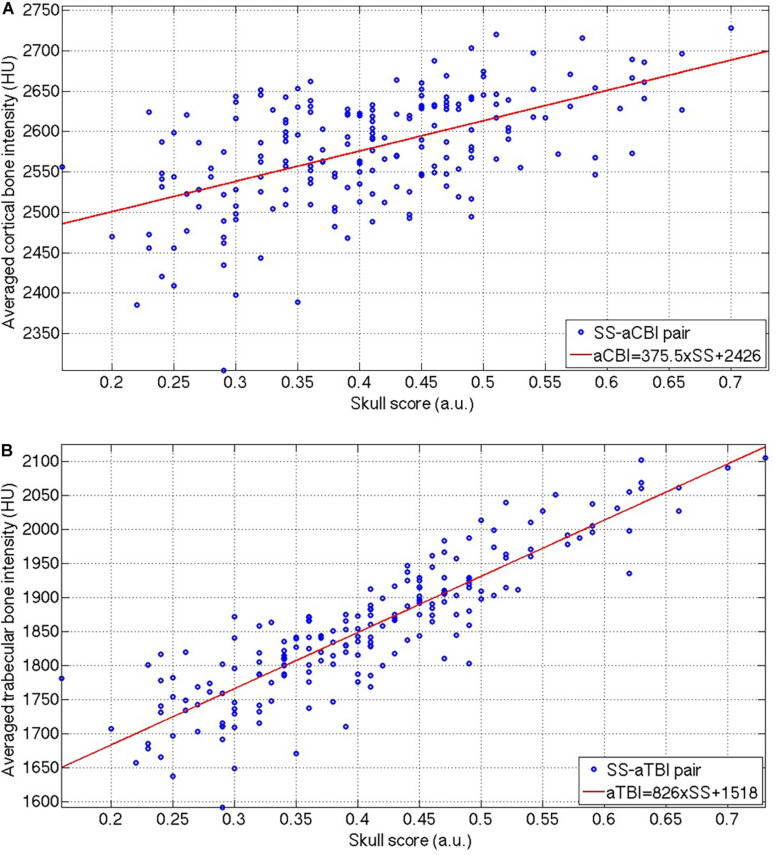
The distribution of the SS-aCBI **(A)** and SS-aTBI **(B)** pairs, and the respective linear fitting (red lines) of these two pairs. SS, skull score; aCBI, average cortical bone intensity; aTBI, average trabecular bone intensity.

Figures 4A,B show that the aCBI and aTBI were linearly correlated with SS, and the linear relations were:

(4) a⁢C⁢B⁢I=375.5×S⁢S+2426

(5) a⁢T⁢B⁢I=826×S⁢S+1518

between aCBI and aTBI to SS, respectively. However, the aCBI was less correlated (*R* = 0.5723) to SS than that of aTBI to SS (*R* = 0.8842). Moreover, the slope of the linear relation between the aTBI and the SS was higher (Eq. 5, slope = 826) than that between the aCBI and the SS (Eq. 4, slope = 375.5).

## Discussion

This study demonstrated the distribution of SS in the population of tremor dominant patients in Taiwan. Only half of the patients fulfilled the empirical SS criteria for the MRgFUS treatment. To our knowledge, this is the first report of SS distribution in patients with tremors.

In this study, we screened 246 patients, and ET patients occupied about 46% (114/246) of total participants. This result was consistent with the perception that ET is the most common movement disorder compared to others (Thanvi et al., 2006) in elderly people. However, the average age of the participants enrolled in this study was high and this limitation might affect the following analysis of the SS or SDR distributions.

Traditional SDR calculation is done by first defining the regions of cortical bone and trabecular bone of the skull and then dividing the average trabecular image intensity by the average cortical image intensity. Our approach differed, and the SS, which is offered by InSightec Inc., was calculated by averaging across all the ratios of the minimum and the maximum of the HU number of the skull CT image along thousands of spatial pathways from the ultrasound transducer to the simulated target in the subcortical area. The traditional SDR is in practice the same as the cSDR in this study in terms of the calculation, which is the averaged tubercular bone intensity over the averaged cortical bone intensity. We used our customized algorithm to automatically extract the skull which has a similar skull region for the SS calculation and then calculate the traditional SDR. Compared with the SS, our cSDR calculation is free from complex steps when calculating the SS.

A high SS suggested a high possibility of success to the MRgFUS thalamotomy to relieve the tremor (Chang et al., 2016), However, according to the SS distribution from our results (Figure 2B), nearly half of the patients have an SS lower than 0.4 in tremor patients of Taiwanese descent, indicating that for those patients, it might be difficult to raise the temperature to the therapeutic 54-degree during the MRgFUS thalamotomy. It is noteworthy that there was a closed 20% of the patients with SS lower than 0.3, indicating the bare success of the MRgFUS thalamotomy for those patients. It minimizes the feasibility of treatment in Asia.

Interestingly, SS is correlated with the trabecular bone rather than the cortical bone. The bootstrapped samples of the SS-cSDR pairs showed a high correlation between SS and cSDR (Figure 3), and the value of the SS was always lower than cSDR in our data. Theoretically, the highest value in the SS calculation indicated the intensity of the cortical bone while the lowest value might not necessarily come from the trabecular bone. The lowest value may represent the intensity of the trabecular bone space or the partial volume of the trabecular bone. The larger difference between the highest and lowest value would lead to a smaller SS. In comparison, our cSDR calculation only use the trabecular bone value, and it would decrease the difference to the cortical bone, leading to a larger cSDR value than the SS. Moreover, according to Eq. (3), SS was more sensitive than cSDR to the cortical and trabecular bone differences since 1 SS would only produce 0.8466 SDR. In Figures 4A,B, both the aTBI and the aCBI have a linear correlation with the SS, and the slope of the respective linear fitting indicating that the aTBI increased higher than the aCBI when increasing the SS, and vice versa. This might be due to the fact that the CBI has smaller variation across patients than the TBI. Here, our data showed a reliable relation of cSDR with SS. Moreover, a possible solution for low SDR should be focused on the aTBI rather than aCBI.

## Conclusion

To our knowledge, this is the first report of the SS population for the MRgFUS thalamotomy in Taiwanese tremor patients. Our results suggest that in nearly half of the Taiwanese patients with tremors it might not be feasible to achieve the therapeutic temperature using current MRgFUS thalamotomy. Accordingly, it is crucial to improve the energy-temperature efficiency for the MRgFUS thalamotomy.

Moreover, our results showed a linear relationship between the SS and our cSDR. In addition, SS correlated more to the trabecular bone than cortical bone. Clinically, seeking a medical approach to increase the aTBI intensity might increase the SS, and further improve the energy-temperature efficiency during the MRgFUS treatment.

## Data Availability Statement

The original contributions presented in the study are included in the article/supplementary material, further inquiries can be directed to the corresponding author.

## Ethics Statement

The studies involving human participants were reviewed and approved by the Institutional Review Board, Show Chwan Memorial Hospital. The patients/participants provided their written informed consent to participate in this study.

## Author Contributions

KW-KT and J-CC proposed the research idea and wrote the manuscript. H-CL collected the clinical data. W-CC provided the clinical suggestions. TT and JC supported the literature review and helped to revise the manuscript. C-YW supported the data analysis and prepared the manuscript for submission. All authors read and approved the final manuscript.

## Conflict of Interest

The authors declare that the research was conducted in the absence of any commercial or financial relationships that could be construed as a potential conflict of interest.
